# Impact of missing participant data for dichotomous outcomes on pooled effect estimates in systematic reviews: a protocol for a methodological study

**DOI:** 10.1186/2046-4053-3-137

**Published:** 2014-11-26

**Authors:** Elie A Akl, Lara A Kahale, Arnav Agarwal, Nada Al-Matari, Shanil Ebrahim, Paul Elias Alexander, Matthias Briel, Romina Brignardello-Petersen, Jason W Busse, Batoul Diab, Alfonso Iorio, Joey Kwong, Ling Li, Luciane Cruz Lopes, Reem Mustafa, Ignacio Neumann, Kari AO Tikkinen, Per Olav Vandvik, Yuqing Zhang, Pablo Alonso-Coello, Gordon Guyatt

**Affiliations:** Department of Internal Medicine, Clinical Epidemiology Unit, American University of Beirut Medical Center, PO Box: 11–0236, Riad-El-Solh, Beirut 1107, 2020 Beirut, Lebanon; Department of Clinical Epidemiology and Biostatistics, McMaster University, Hamilton, Canada; Basel Institute for Clinical Epidemiology and Biostatistics, University Hospital of Basel, Hebelstrasse 10, 4031 Basel, Switzerland; Evidence Based Dentistry Unit, Faculty of Dentistry, Universidad de Chile, Santiago, Chile; Department of Anesthesia, McMaster University, Hamilton, Canada; The Michael G. DeGroote Institute for Pain Research and Care, McMaster University, Hamilton, Canada; Pharmaceutical Sciences Post graduate Course, University of Sorocaba, UNISO, Sorocaba, Sao Paulo, Brazil; Stanford Prevention Research Center, Department of Medicine, Stanford University, Stanford, USA; Clinical Research and Evaluation Unit, Chinese Evidence-based Medicine Center, West China Hospital, Sichuan University, Chengdu, China; Departments of Medicine and Biomedical and Health Informatics, University of Missouri, Kansas City, Kansas USA; Department of Internal Medicine, School of Medicine, Pontificia Universidad Católica de Chile, Santiago, Chile; Departments of Urology and Public Health, Helsinki University Central Hospital and University of Helsinki, Helsinki, Finland; Norwegian Knowledge Centre for the Health Services, Oslo, Norway; Department of Medicine, Innlandet Hospital Trust, Gjøvik, Norway; Iberoamerican Cochrane Centre, Institute of Biomedical Research (IIB Sant Pau), Barcelona, Spain; Department of Medicine, McMaster University, Hamilton, Canada

**Keywords:** Missing participant data, Imputation, Risk of bias, Trials, Systematic reviews, Meta-analysis

## Abstract

**Background:**

There is no consensus on how authors conducting meta-analysis should deal with trial participants with missing outcome data. The objectives of this study are to assess in Cochrane and non-Cochrane systematic reviews: (1) which categories of trial participants the systematic review authors consider as having missing participant data (MPD), (2) how trialists reported on participants with missing outcome data in trials, (3) whether systematic reviewer authors actually dealt with MPD in their meta-analyses of dichotomous outcomes consistently with their reported methods, and (4) the impact of different methods of dealing with MPD on pooled effect estimates in meta-analyses of dichotomous outcomes.

**Methods/Design:**

We will conduct a methodological study of Cochrane and non-Cochrane systematic reviews. Eligible systematic reviews will include a group-level meta-analysis of a patient-important dichotomous efficacy outcome, with a statistically significant effect estimate. Teams of two reviewers will determine eligibility and subsequently extract information from each eligible systematic review in duplicate and independently, using standardized, pre-piloted forms. The teams will then use a similar process to extract information from the trials included in the meta-analyses of interest. We will assess first which categories of trial participants the systematic reviewers consider as having MPD. Second, we will assess how trialists reported on participants with missing outcome data in trials. Third, we will compare what systematic reviewers report having done, and what they actually did, in dealing with MPD in their meta-analysis. Fourth, we will conduct imputation studies to assess the effects of different methods of dealing with MPD on the pooled effect estimates of meta-analyses. We will specifically calculate for each method (1) the percentage of systematic reviews that lose statistical significance and (2) the mean change of effect estimates across systematic reviews.

**Discussion:**

The impact of different methods of dealing with MPD on pooled effect estimates will help judge the associated risk of bias in systematic reviews. Our findings will inform recommendations regarding what assumptions for MPD should be used to test the robustness of meta-analytical results.

**Electronic supplementary material:**

The online version of this article (doi:10.1186/2046-4053-3-137) contains supplementary material, which is available to authorized users.

## Background

Missing data for the outcomes of interest is a common problem in randomized trials[1]. In one study, almost one in every three trials with statistically significant results lost statistical significance when making plausible assumptions about the outcomes of participants with missing data[1]. This reduces our confidence in the effect estimates resulting not only from these trials but also from systematic reviews including them.

One challenge with abstracting data from trial reports is understanding whether or not data from a specific category of participants is missing. For example, a trial report might indicate that a certain number of participants withdrew consent, without indicating whether or not they continued to be followed-up. In these cases, systematic review authors might need to make assumptions based on their best guess.

Moreover, a recent methodological survey found that systematic reviews are deficient in terms of dealing with and assessing the risk of bias associated with missing participant data (MPD) (unpublished data by Akl et al.)[2]. The Cochrane Collaboration’s Risk of Bias (RoB) tool was designed to help in assessing bias associated with a number of factors, including MPD[3]. In a recent study evaluating stakeholders’ experiences with, and perceptions of, the Cochrane RoB tool participants cited incomplete outcome data as one of the most difficult domains to assess[4]. They also requested more guidance on how to incorporate RoB assessments into meta-analyses and conclusions[4].

One approach to assessing the extent of risk of bias associated with MPD is to conduct sensitivity analyses based on different assumptions regarding the outcomes of participants with missing outcome data[5–7]. Examples of these assumptions include none, and all or a specified proportion of participants in each group suffering the outcome of interest. No study has thus far assessed how systematic reviewers actually deal with MPD. Similarly, there is a lack of evidence about the impact of different approaches for dealing with MPD on pooled effect estimates. This leaves uncertain the extent to which results of systematic reviews are vulnerable to MPD.

### Objectives

The objectives of this study are to assess in Cochrane and non-Cochrane systematic reviews: (1) which categories of trial participants the systematic review authors consider as having MPD, (2) how trialists reported on participants with missing outcome data in trials, (3) whether systematic reviewer authors actually dealt with MPD in their meta-analyses of dichotomous outcomes consistently with their reported methods, and (4) the impact of different methods of dealing with MPD on pooled effect estimates in meta-analyses of dichotomous outcomes.

## Methods/Design

We did not register this protocol with PROSPERO given the register does not accept methodological reviews.

### Definitions

MPD refers to outcome data for trial participants that are not available to the systematic reviewer authors (from the published trial reports or personal contact with trial authors) for inclusion in their meta-analyses.

MPD do not relate to any of the following:

- Missing studies (e.g., unpublished studies);
- Entire unreported outcomes (e.g., outcomes planned in trial protocols but not included in trial reports).

Cochrane systematic reviews are defined as systematic reviews published in the Cochrane Database of Systematic Reviews. All other systematic reviews will be considered non-Cochrane systematic reviews.

A patient-important outcome is defined as an outcome for which one would answer with “yes” to the following question: “if the patient knew that this outcome was the only thing to change with treatment, would the patient consider receiving this treatment if associated with burden, side effects, or cost?” We will use a previously developed hierarchy of outcomes for the selection of one outcome of interest (Additional file1)[8]. Categories I, II, and III include patient-important outcomes. Category IV includes surrogate outcomes, which are not considered as patient-important. For a composite outcome to be considered as patient-important, we will require all of its components to be patient-important[8].

### Categories of participants with potential MPD

We will use the following mutually exclusive categories of participants that could be potentially counted as having MPD, at the trial level: “mistakenly randomized and inappropriately excluded,” “did not receive any treatment,” “withdrew consent,” “outcome not assessable,” “dead,” “experienced adverse events,” “non-compliant,” “discontinued prematurely,” “cross-over,” “moved out of country,” and “lost to follow-up” (lost to follow-up for reasons not considered in our other categories). When it is not clear whether the participants in those categories have MPD or not, we will assume the following:

- “Mistakenly randomized and inappropriately excluded,” and “did not receive any treatment” participant categories, which are defined prior to the initiation of the trial intervention, most likely have MPD;
- “Withdrew consent,” “outcome not assessable,” “moved out of country,” and “lost to follow-up” (for reasons not considered in our other categories) participant categories, which are defined after the initiation of the trial intervention, most likely have MPD;
- “Dead,” “experienced adverse events,” “non-compliant,” “discontinued prematurely,” and “cross-over” most likely do not have MPD.

The number of participants with missing data will be the sum of the number of participants in each of the categories of participants known or inferred to have missing data.

### Eligibility criteria

The inclusion criteria for a systematic review:

1. Meets the following minimum criteria we set for a systematic review of trials:
  - Described as “systematic review” and/or “meta-analysis” of trials;
  - Compares a clinical intervention to another (or to no intervention);
  - Reports a search strategy of at least one database;
2. Addresses a preventive or a therapeutic clinical question in humans (diagnostic, prognostic, public health, and health service questions are not eligible);
3. Is either a Cochrane review or a non-Cochrane review published in one of the core clinical journals;
4. Includes a meta-analysis that meets the following criteria:
  - Is a group-level meta-analysis of randomized controlled trials and/or controlled clinical trials (e.g., network meta-analysis, Bayesian meta-analysis, and meta-regression are not eligible);
  - Reports an effect estimate expressed as a dichotomous measure (including relative risk (RR) or odds ratio (OR); excluding those produced by generic inverse variance method);
  - Reports a statistically significant pooled effect estimate from at least two trials for a patient-important efficacy outcome; statistical significance refers to *p* value < 0.05 or confidence interval (CI) not including 1.0.

The exclusion criteria are:

1. A systematic review that is a duplicate publication (e.g., a Cochrane systematic review published in both the Cochrane Library and in a peer-reviewed journal)
2. A meta-analysis with more than 20 included trials, for feasibility purposes.
3. A meta-analysis not reporting the numerical data used or each included trial (i.e., numerator and denominator per arm).

### Search strategy

We will search for Cochrane systematic reviews in the Cochrane Library. We will use the Ovid MEDLINE interface to search for non-Cochrane systematic reviews in the Core Clinical Journals (119 English language clinical journals indexed under Abridged Index Medicus by the National Library of Medicine (available at http://www.nlm.nih.gov/bsd/aim.html). The Abridged Index Medicus was initiated in 1970 to enable direct access to selected biomedical journals of interest to practicing physicians. We will restrict the search to 1 year but will not impose any language restrictions. Additional file2 provides the details of the search strategy.

### Random sampling of citations

We will retrieve two random samples of Cochrane and non-Cochrane systematic reviews from the pool of citations identified by our search. We will screen these two samples for eligibility using the above criteria. We will repeat the random sampling process as needed until reaching the final sample size, which will include the same number of Cochrane and non-Cochrane systematic reviews (see Sample size section).

### Selection process

We will conduct title and abstract screening, full-text screening, and data abstraction in teams of two reviewers working independently and in duplicate. At the title and abstract screening stage, we will obtain the full text for any citation included by at least one reviewer. At the full-text screening and data abstraction stages, the reviewers will resolve discrepancies by consensus, and if unsuccessful, with the help of a third reviewer. We will carry out calibration exercises at each level of the process for the purpose of verifying the validity and consistency of the review process. We will also develop and pilot test standardized data abstraction forms with instructions. A core group will communicate regularly to discuss progress and potential difficulties. A study flow will be developed to describe the results of the different steps of the selection process.

If a systematic review reports on more than one pair-wise comparison with eligible meta-analyses, we will select the first one reported in the main text. If the selected comparison includes more than one eligible meta-analysis, we will select the one with the outcome that ranks the highest on the outcome hierarchy (Additional file1). If more than one outcome ranks the same on the outcome hierarchy, we will select the one reported first in the main text.

### Data abstraction

We will conduct data abstraction using web-based systematic review software (DistillerSR™). In a first phase, we will collect data from the eligible systematic reviews and the eligible meta-analyses. In the second phase, we will collect data from trials included in the eligible meta-analyses.

Phase 1: we will abstract the following data from each eligible systematic review:

Characteristics of the systematic review:

- Type of systematic review (i.e., Cochrane vs. non-Cochrane);
- Use or non-use of the Grading of Recommendations, Assessment, Development, and Evaluation (GRADE) approach for assessing the confidence in effect estimates by outcome;[9]
- Source of funding (e.g., private for profit, private not for profit, governmental, not funded, not reported).

Characteristics of the comparison of interest:

- Type of intervention and control (e.g., pharmacological, surgical);
- Outcome ranking on patient-importance hierarchy (i.e., I, II, III);
- Duration of follow-up for outcome measurement;
- Type of outcome (e.g., efficacy, safety).

Missing participant data:

In reference to the comparison and outcome addressed in the eligible meta-analysis, we will verify whether the systematic review:

- Documented the categories of participants that could potentially be counted as having MPD (see previous section “Categories of participants with potential MPD”), and when documented, the number within each category;
- Reported number of MPD and at what level (e.g., at the study arm level, study level, across studies);
- Explicitly stated using the following for their meta-analysis: intention-to-treat, modified intention-to-treat, per protocol, or as treated;
- Explicitly stated analyzing participants in the group to which they were randomized;
- Explicitly stated the analytical method for dealing with MPD in the eligible meta-analysis (i.e., to account for MPD when generating the best effect estimate): complete case analysis, making assumptions for MPD, using the assumption used by the trialists, or excluding trials with high rate of MPD. Tables 1 and2 present the numerical details of the different assumptions that could be used to deal with MPD;

**Table 1:** **Numerical information from each trial to be used in the imputation analyses**

	Intervention arm			Control arm		
	Number of participants randomized	Number of participant with missing data	Number of observed events	Number of participants randomized	Number of participant with missing data	Number of observed events
Number of trial participants	*a*	*b*	*c*	*e*	*f*	*g*

The number of participants with missing data is the sum of the number of participants in each of the categories of participants known or assumed to have missing data (see section “Categories of participants with potential MPD”).

**Table 2:** **Numerical details of different methods to be used in the imputation analyses**

Analytic method	Intervention arm		Control arm	
	Numerator	Denominator	Numerator	Denominator
Complete (available) case analysis	*c*	*a* - *b*	*g*	*e* - *f*
None has event	*c*	*a*	*g*	*e*
All had event	*b* + *c*	*a*	*f* + *g*	*e*
Best case scenario	*c*	*a*	*f* + *g*	*e*
Worst case scenario	*b* + *c*	*a*	*g*	*e*
Using the concept of RI_LTFU/FU_	[*b x y x c*/(*a* - *b*)] + *c*	*a*	[*f x z x g*/(*e* - *f*)] + *g*	*e*
Incidence for missing participants same as observed in same arm^a^	[*b x c*/(*a* - *b*)] + *c*	*a*	[*f x g*/(*e* - *f*)] + *g*	*e*
Incidence for missing participants in both arms same as observed in control arm	[*b x g*/(*e* - *f*)] + *c*	*a*	[*f x g*/(*e* - *f*) ] + *g*	*e*

Refer to Table 1 for what values letters a-g refer to *y* and *z* refer to RI_LTFU/FU_ in the intervention and control arm, respectively[8].

^a^This is a special case of RI_LTFU/FU_ method where *y* = *z* = 1.

- Explicitly stated any additional meta-analyses for dealing with MPD for the outcome of interest (“reported analytical method”) (i.e., to judge the risk of bias associated with MPD): complete case analysis, making assumptions for MPD, using the assumption used by the trialists, or excluding trials with high rate of MPD (see Tables 1 and2);
- Explicitly stated using assumptions for MPD accounted for uncertainty associated with imputing events;
- Justification for the analytical method used to deal with MPD in the eligible meta-analysis;
- Tool(s) used to judge risk of bias associated with MPD at the study level (e.g., Cochrane RoB tool), if any;
- Method(s) used to assess risk of bias associated with MPD at the meta-analysis level (e.g., sensitivity analysis, subgroup analysis), if any.

Data from the eligible meta-analysis:

- Number of trials included;
- Numerator and denominator used in the meta-analysis for each arm for each trial;
- Number of MPD reported (per arm or combined);
- Pooled relative effect measure (RR or OR) and its associated CI, *p* value, and measure of heterogeneity (*I*^2^);
- Analysis model used (i.e., random effect or fixed effect);
- Statistical method used (e.g., Mantel-Haenszel or Peto);
- Results of sensitivity analyses applied to account for MPD.

Phase 2: we will abstract the following data from all trials included in each eligible meta-analysis:

Characteristics of the trials:

- Type of reference (e.g., abstract, full-text article);
- Type of trial (e.g., randomized controlled trial, controlled clinical trial);
- Duration of follow-up for outcome measurement;
- Source of funding (e.g., private for profit, private not for profit, governmental, not funded, not reported);
- Type of outcome (e.g., efficacy, safety).

Missing participant data:

- Presence of MPD;
- Whether the trialists report the MPD for this specific outcome, as opposed to reporting premature end of follow-up for trial participants in general;
- Whether the trialists followed-up categories of participants that could be potentially counted as having MPD (see previous section “Categories of participants with potential MPD”);
- Whether the trialists included in the analysis the categories of participants that could be potentially counted as having MPD in the analysis;
- Level of reporting number of participants with MPD (e.g., per arm, both arms combined);
- Whether the trialists described the pattern of missingness;
- Whether the trialists compared the baseline characteristics of participants with and without MPD (e.g., MPD group vs. non-MPD group, MPD first arm vs. MPD second arm)
- Number of participants in each of the categories of participants that, following our rules stated previously, would be counted as having MPD, per arm;
- Whether the trialists report using the following for their analysis: intention-to-treat, modified intention-to-treat, per protocol, or as treated;
- Whether the trialists report analyzing participants in the group to which they were randomized;
- Whether the trialists report the analytical method for dealing with MPD in the main analysis, i.e., to account for MPD when generating the effect estimate (e.g., complete case analysis, making assumptions for MPD);
- Whether methods using assumptions for MPD accounted for uncertainty associated with imputing events;
- Whether the trialists provided any justification for the method used to deal with MPD in the main analysis;
- Method(s) used to assess risk of bias associated with MPD in the analysis (e.g., sensitivity analysis, different methods for different subgroups of participants with MPD), if any;
- Numerical information for the comparison and outcome of interest: number randomized per arm, number of observed events per arm (see Table 1);
- Relative effect measure (e.g., RR, OR) and its associated CI, and *p* value;
- Analytical method “actually” used by the systematic review authors based on the numerical information provided by the trialists;
- Whether the trialists report any additional analytical methods for dealing with MPD (“reported analytical method”), i.e., to judge the risk of bias associated with MPD (e.g., complete case analysis, making assumptions for MPD);
- Whether the trialists incorporated implications of MPD in results or discussion sections.

### Sample size

The sample size will be based on the goal of achieving enough precision (CI width of 0.1) around the proportion of systematic reviews (both Cochrane and non-Cochrane) for which the pooled effect estimate crosses the boundary of conventional statistical significance (further details below under “Impact of different methods of dealing with MPD on pooled effect estimates”). We will use the findings after including the first 100 systematic reviews to calculate the final sample size. We will include equal numbers of Cochrane and non-Cochrane systematic reviews.

### Data analysis

#### Agreement

We will assess agreement between reviewers for inclusion of systematic reviews at the full-text screening stage for their first judgment (i.e., before the step of reaching agreement by consensus) using chance-corrected agreement (kappa statistic). If fewer than 15% or more than 85% of citations are included in this study, we will measure agreement using chance-independent agreement (phi statistic)[10]. We will interpret the agreement statistics using the guidelines proposed by Landis and Koch[11]: kappa values of 0 to 0.20 represent slight agreement, 0.21 to 0.40 fair agreement, 0.41 to 0.60 moderate agreement, 0.61 to 0.80 substantial agreement, and greater than 0.80 almost perfect agreement. We will use these same thresholds for interpreting phi.

#### Characteristics of the included systematic reviews

We will conduct a descriptive analysis of the characteristics of included systematic reviews and the comparisons of interest. For all descriptive analyses, we will use percentages for dichotomous variables. To describe the distribution of continuous variables, we will use the mean and standard deviation when distribution is near normal and median and interquartile range (IQR) when the distribution is substantially skewed. We will use the Shapiro-Wilk test to evaluate whether the distributions of continuous variables violate assumptions of normality.

For all relevant analyses, we will test for the statistical significance of the differences between the Cochrane and non-Cochrane reviews. For dichotomous variables, we will use the chi-square test. For continuous variables, we will use Student’s two independent sample *t*-test for two independent samples when the distribution is normal and the Mann–Whitney *U*-test (a non-parametric test for two independent samples) when the distribution is not normal. When results differ, we will present the results stratified by the systematic review type (i.e., Cochrane vs. non-Cochrane) and consider presenting the overall results as well. When results do not differ, we will focus on the overall results but consider presentation of stratified results as well.

#### Categories of trial participants reviewers considered as having MPD

For each category of participants that could be potentially counted as having MPD (refer to section above on “Categories of participants with potential MPD”), we will calculate:

- The percentage of systematic reviews documenting that category;
- The percentage of trials documenting that category;
- The distribution across trials of the proportion of participants belonging to that category.

We will compare (1) data from the trial regarding the number of participants in the different categories of participants that, following our rules stated previously, would be counted as having MPD, to (2) the number of participants considered in the systematic review as having MPD. We will conduct this analysis irrespective of what categories of participants that could be potentially counted as having MPD are reported by the systematic review.

#### Reporting of MPD by trialists

We will conduct a descriptive analysis of the reporting of MPD by trialists. We will calculate:

- The percentage of trials following-up categories of participants that could be potentially counted as having MPD;
- The percentage of trials documenting the number of participants in each arm with MPD; if applicable;
- The percentage of trials documenting different numbers of participants with MPD across different outcomes, if applicable;
- The percentage of trials documenting the pattern of missingness;
- The percentage of trials documenting the comparison of the baseline characteristics of participants with and without missing participant data;
- The percentage of trials documenting the methods used to assess risk of bias associated with MPD in the analysis.

#### Reported vs. actual method of dealing with of MPD in meta-analysis

First, we will conduct the following descriptive analysis:

- The percentage of systematic reviews using each of the terms “intention-to-treat,” “modified intention-to-treat,” “per protocol,” and “as treated;”
- The percentage of systematic reviews reporting a plan to analyze participants in the group to which they were randomized;
- The percentage of systematic reviews using different analytical methods for dealing with MPD in the main analysis, i.e., when generating the best effect estimate;
- The percentage of systematic reviews using imputed data for MPD and accounting for uncertainty associated with imputing events;
- The percentage of systematic reviews justifying the analytical method used to deal with MPD in the eligible meta-analysis;
- The percentage of systematic reviews using different analytical methods (e.g., sensitivity analysis, subgroup analysis) to judge risk of bias associated with MPD;
- The percentage of systematic reviews using different tools (e.g., Cochrane RoB tool) to measure risk of bias associated with MPD in individual trials, and for the body of evidence, across trials, supporting the outcome of interest.

Second, we will analyze the “actually used analytical method” for dealing with MPD. For this purpose, and for each trial included in the eligible meta-analysis, we will compare data from the trial report (number randomized, number of observed events, number with missing data) to the corresponding data included in the meta-analysis (numerator, denominator). Based on these comparisons, we will categorize the “actually used analytical method” for dealing with MPD as: complete case analysis, making assumptions for MPD, using the assumption used by the trialists, or excluding trials with high rate of MPD.

We will develop and pilot test specific rules for categorizing the “actually used analytical method” for dealing with MPD. As part of this process, we will note when some of these data appear to have been wrongly abstracted. Two reviewers will make these judgments in duplicate and independently. They will resolve discrepancies by consensus, and if unsuccessful, with the help of a third reviewer.

Third, we will conduct a bivariate analysis to assess the association between the “actually used analytical method” and the following characteristics:

- Whether the trialists report using the following for their analysis: intention-to-treat, modified intention-to-treat, per protocol, as treated;
- Whether the trialists report analyzing participants in the group to which they were randomized.

Fourth, we will compare the “actually used analytical method” for dealing with MPD with the “reported analytical method.” The latter refers to the method the authors report for dealing with MDP (e.g., complete case analysis, making assumptions for MPD, using the assumption used by the trialists, or excluding trials with high rate of MPD). We will calculate the percentage of systematic reviews with “discrepancy” between “reported” and “actually used” analytical methods. We will display the results in a matrix with “reported” analytical methods displayed in columns and “actually used” analytical methods displayed in rows. We will test whether the distribution of studies according to these two variables is due to chance using the chi-square statistical test.

Finally, we will conduct a multivariable logistic regression analysis using the “discrepancy” as the dependent variable and the general characteristics of the included systematic reviews as the independent variables: type of systematic review (i.e., Cochrane vs. non-Cochrane), use of GRADE, source of funding, type of intervention, and outcome patient-importance ranking.

#### Impact of different methods of dealing with MPD on pooled effect estimates

For the following analyses, we will use “original effect estimate” to refer to the effect estimate reported in the eligible meta-analyses. For each eligible meta-analysis, we will first attempt to reproduce the original analysis. When this analysis generates a different effect estimate that is not statistically significant, we will exclude the corresponding meta-analysis from this part of the study.

Then, we will apply a number of methods of dealing with MPD (see below). Each of these methods will generate a set of values for the numerator and denominator in each arm of each trial included in the meta-analysis. We will use these values to conduct the sensitivity analyses using the same pooled relative effect measure (RR or OR), the same analysis model (random effect or fixed effect), and the same statistical method (e.g., Mantel-Haenszel, Peto) used by the systematic review authors to generate the original effect estimate. We will use the “assumption pooled effect estimates” to refer to the results of those sensitivity analyses. For outcomes that have a measure of effect greater than 1.00, we will reverse the direction of the comparison at the time of the analysis.

In terms of applying different methods of dealing with MPD, we will start by conducting a complete case analysis. As described in detail in the subsequent paragraphs, we will also apply both “implausible” (but sometimes used) and more plausible assumptions for dealing with MPD. We will apply to each of these assumptions and statistical approaches to take uncertainty into account.

We will use the following implausible assumptions,[5] described in more details in Table 2:

- None of the participants with MPD had the event;
- All participants with MPD had the event;
- None of the participants with MPD in the treatment group had the event and all participants with MPD in the control group did (best case scenario);
- All participants with MPD in the treatment group had the event and none of participants with MPD in the control group did (worst case scenario).

We will also use the more plausible assumptions that the event incidence among participants with missing data is equal to or higher than the observed event incidence among followed-up and by a specified ratio (see Table 2). For this purpose, we define RI_NotFU/FU_ as the relative event incidence among those not followed-up relative to the event incidence among those followed-up. We will test assumptions combining a range of plausible RI_NotFU/FU_ values (1, 1.5, 2, 3, and 5) in the two comparison arms. This will follow the methodology our group used to conduct a similar project focusing on trials (the LOST-IT project)[8]. In addition, we will use the assumption that the incidence for missing participants in both arms is the same as the one observed in the trial control arm.

Making assumptions implies imputing the outcomes of participants with missing data. That would increase the total number of events and result in inappropriate narrowing the CI of the effect estimate. In order to avoid such inappropriate narrowing of the CI, we will apply statistical approaches to take into account the uncertainty associated with imputing outcomes. One recommended approach consists of using a “variance inflation factor” to inflate the standard errors or variances of effect estimates[12, 13].

For each of the assumptions, we will calculate the proportion of systematic reviews for which the “assumption pooled effect estimate” crosses the boundary of conventional statistical significance.

## Discussion

The main objective of this study is to assess in Cochrane and non-Cochrane systematic reviews the impact of different methods of dealing with MPD on pooled effect estimates of dichotomous outcomes, with focus on systematic reviews reporting statistically significant findings. We will also compare the number of participants that following our rules would be counted as having MPD with the number of participants considered in the systematic review counted as having MPD.

The strengths of our proposed study include the adoption of systematic and transparent methods, including specific and explicit eligibility criteria, sensitive search strategies, and duplicate and independent processes for study selection, data abstraction, and data interpretation (e.g., judgment about the “actually used” analytical methods). Also, we will use standardized and pilot tested forms supplemented with specific and detailed instructions. We will explore issues that have not previously been addressed, particularly in terms of the impact of MPD on pooled effect estimates. Finally, our group has extensive experience in completing methodological studies involving large samples[1, 14–18].

The major limitation of our study is the need for reviewers’ judgments at different stages of the process (e.g., judgment about the “actually used” analytical methods). The specific and detailed instructions pilot testing, and calibration exercises described previously should help minimize disagreement.

This study will focus on dichotomous outcome data, given the methodological and statistical issues vary substantively for continuous data. Also, it will focus on systematic reviews with a statistically significant pooled effect estimate for a patient-important efficacy outcome because they are the most likely to influence clinical practice.

We will be assessing how Cochrane and non-Cochrane systematic reviews compare across the three objectives of this study. A major reason is that the former reviews tend to be of higher methodological quality compared to the latter[19, 20]. Indeed, we opted to assess the quality of the included systematic reviews through using two indicators: the type of systematic review (Cochrane vs. non-Cochrane) and the use of the GRADE approach. In a recently conducted methodological survey on MPD in systematic review, regression analyses identified these two factors, but not the AMSTAR tool,[11] as statistically significant predictors of two MPD related variables (i.e., whether the systematic review judged the risk of bias associated with MPD and whether the systematic review used complete case analysis) (unpublished data).

For the third objective of this study, and in addition to assessing the impact of plausible methods of dealing with MPD, we will assess the impact of implausible methods. This is because these implausible methods (e.g., none had the event, all had the event) are sometimes used in published meta-analyses. Also, the worst case scenario could be used to test the robustness of the results[5].

Understanding which categories of trial participants the systematic review authors consider as having MPD and how they deal with MPD in meta-analysis will help in better defining standards for conducting and reporting systematic reviews. Assessing the impact of different methods of dealing with MPD on pooled effect estimates will help judge the associated risk of bias in systematic reviews. Our findings will inform recommendations regarding what assumptions for MPD should be used to test the robustness of the meta-analytical results. They will also help in better defining how to assess risk of bias associated with MPD at the systematic review level and provide insight into the frequency with which MPD compromises trust in statistically significant meta-analytical results. Finally, the publication of this protocol will contribute to making the objectives and design of methodological surveys more transparent[2, 8, 21–24].

## Electronic supplementary material

Additional file 1:**Hierarchy of outcomes relative to patient importance.** The hierarchy of outcomes is used for the selection of the outcome of interest. Categories I, II, and III include patient-important outcomes. Category IV includes surrogate outcomes, which are not considered as patient-important. (DOCX 15 KB)

Additional file 2:**Search strategy.** Search strategy for non-Cochrane reviews, using Ovid MEDLINE (R) In-Process and Other Non-Indexed Citations and Ovid MEDLINE (R) <1946 to Present>. (DOCX 14 KB)

## Abbreviations

MPD: missing participant data
RoB: risk of bias
RR: relative risk
OR: odds ratio
CI: confidence interval
RCT: randomized controlled trial
CCT: controlled clinical trial
IQR: interquartile range
RI_NotFU/FU_: the relative event incidence among those not followed-up relative to the event incidence among those followed-up
AMSTAR: a measurement tool to assess systematic reviews.
