# Supplementary material for: Impact of missing participant data for dichotomous outcomes on pooled effect estimates in systematic reviews: a protocol for a methodological study
Source: Syst Rev. 2014 Nov 26;3:137. doi: 10.1186/2046-4053-3-137 (PMC4285551; doi:10.1186/2046-4053-3-137)
Supplement: Supplementary file 1 — Additional file 1:Hierarchy of outcomes relative to patient importance. The hierarchy of outcomes is used for the selection of the outcome of interest. Categories I, II, and III include patient-important outcomes. Category IV includes surrogate outcomes, which are not considered as patient-important. (DOCX 15 KB) [file 13643_2014_306_MOESM1_ESM.docx]

**Additional file 1:** Hierarchy of outcomes relative to patient importance

| 1. Mortality    1. all - cause mortality    2. disease specific mortality 2. Morbidity    1. cardiovascular major morbid events    2. other major morbid events (e.g. loss of vision, seizures, fracture, revascularization)    3. onset/recurrence/relapse/remission of cancer and other chronic diseases (e.g. COPD exacerbation, new onset of diabetes)    4. renal failure requiring dialysis    5. hospitalization, medical and surgical procedures (e.g. placement of a pacemaker, and cardioversion)    6. infections    7. dermatological/ rheumatologic disorders 3. Symptoms/Quality of life/Functional status (e.g. failure to become pregnant, successful nursing/breastfeeding, depression) 4. Surrogate outcomes (e.g. viral load, physical activity, weight loss, cognitive function, recurrent polyps, adherence to medication) |
| --- |
