# Supplementary material for: Impact of missing participant data for dichotomous outcomes on pooled effect estimates in systematic reviews: a protocol for a methodological study
Source: Syst Rev. 2014 Nov 26;3:137. doi: 10.1186/2046-4053-3-137 (PMC4285551; doi:10.1186/2046-4053-3-137)
Supplement: Supplementary file 2 — Additional file 2:Search strategy. Search strategy for non-Cochrane reviews, using Ovid MEDLINE (R) In-Process and Other Non-Indexed Citations and Ovid MEDLINE (R) <1946 to Present>. (DOCX 14 KB) [file 13643_2014_306_MOESM2_ESM.docx]

**Additional file 2:** Search strategy for non-Cochrane reviews, using Ovid MEDLINE(R) In-Process & Other Non-Indexed Citations and Ovid MEDLINE(R) <1946 to Present>:

1 meta analysis.pt.

2 meta anal$.mp.

3 metaanal$.mp.

4 metanal$.mp.

5 meta epidemiolog*.mp.

6 systematic review$.mp.

7 systematic overview$.mp.

8 ((pool: or combined or combining) adj (data or trial* or studies or results)).mp.

9 ((hand adj2 search:) or handsearch:).mp.

10 cochrane.mp.

11 ((quantitative or systematic: or methodologic: or integrative:) adj2 (review: or overview: or synthes: or survey:)).mp.

12 (peto or der simonian or dersimonian).mp.

13 or/1-12

14 (pooled analys: or pooling or mantel haenszel:).mp.

15 fixed effect:.mp.

16 (extraction or medline or embase or pubmed or cinahl).ab.

17 14 or 15 or 16

18 (review: or cochrane).mp.

19 16 and 17

20 13 or 19

21 aim.sb.

22 "2012".yr.

23 20 and 21 and 22
